# A Natural History of the Mammalia

**Published:** 1846-04

**Authors:** 


Art. IV.—*A Natural History of the Mammalia.* By G. R. Waterhouse, Esq., of the British Museum.—*London,* 1845-6. 8vo, Parts I—VI.

The brief title, 'A Natural History of the Mammalia,' led us at first to regard this work as not fairly coming within our province; but on turning to the contents of each number as it has appeared in regular monthly sequence, we have been agreeably surprised to find that we had formed too low an estimate of its value, as the plan includes not only the natural history, but also much connected with the osteology and other structures of the mammalia. The parts already published enter fully into the natural history of the marsupialia, and also into the structure of portions of the skeleton, more especially into the zoological character of the skull and teeth, and the general conformation of the brain. The work thus combines details of high interest to the comparative anatomist as well as to the zoologist, details which ought always to be given together; because, by blending these two kinds of information, the value of each is doubled, and each serves to explain and illustrate the peculiarities of the other.

We are glad to observe that Mr. Waterhouse is enriching his work with facts recently brought to light by Professor Owen respecting the fossil mammalia; and also with others derived from the magnificent accumulation of fossil mammalia under his own care in the British Museum. With these means of knowledge, and with his already well-known extensive acquaintance with the recent species, we are satisfied that his work will prove to be one of considerable value to the anatomist as well as to the zoologist. But we must take leave to criticise his style, which is far too diffuse, and requires condensation—more especially in that part of the work which details the habits and natural history. This is an error into which most zoologists are apt to fall, and to which they are almost necessarily conducted by the very nature of many of the objects they have to describe—objects which are known to be quite distinct from each other, yet which frequently afford so few marked peculiarities that it is only by the minutest details that they can be characterized and described in words. We have no doubt that this hint will suffice. We strongly recommend Mr. Waterhouse to adhere closely to his plan of giving full anatomical details of the skeleton, the teeth, skull, and more especially the conformation of the brain; so that these may be commentaries on the instincts and natural history of the class. In a word, let him give us all *the facts* that can be obtained, but condense his style and mode of communication. We shall then have an exceedingly useful and desirable production.

